# Cryptogenic Organizing Pneumonia and Idiopathic Eosinophilic Pneumonia: A Case Report of Clinically Identical Entities

**DOI:** 10.7759/cureus.40591

**Published:** 2023-06-18

**Authors:** Venkata S Buddhavarapu, Gagandeep Dhillon, Harpreet Grewal, Rahul Kashyap

**Affiliations:** 1 Hospital Medicine, Banner Medical Group, Banner Health, Phoenix, USA; 2 Internal Medicine, University of Maryland Baltimore Washington Medical Center (UM BWMC), Glen Burnie, USA; 3 Radiology, Florida State University College of Medicine, Pensacola, USA; 4 Medicine, Drexel University College of Medicine, Philadelphia, USA; 5 Medicine, Global Clinical Scholars Research Training (GCSRT) Program, Harvard Medical School, Boston, MA, USA; 6 Research, Global Remote Research Program, St Paul, USA; 7 Critical Care Medicine, Mayo Clinic, Rochester, USA; 8 Research, WellSpan Health, York, USA

**Keywords:** acute respiratory distress syndrome (ards), diffuse interstitial lung disease, idiopathic eosinophilic pneumonia, acute eosinophilic pneumonia, cryptogenic organizing pneumonia

## Abstract

Cryptogenic organizing pneumonia (COP) and idiopathic eosinophilic pneumonia (IEP) are two forms of diffuse interstitial lung diseases (ILD) that lead to a rapid respiratory decline in young patients. Both conditions presented with similar clinical and radiological findings, making a clinical diagnosis challenging. They are both considered diagnoses of exclusion, and the treatment for both conditions is high-dose corticosteroids, leading to a quick recovery. Pathological specimens are often required prior to initiating appropriate treatment, leading to significant delays in appropriate therapy and a poorer prognosis. In this case report, we suggest that clinical pearls can be used to establish either diagnosis earlier, which leads to earlier treatment and better outcomes. Our patient presented with an acute respiratory distress syndrome (ARDS) picture, bilateral interstitial infiltrates with peripheral predominance, eosinophilia, and a negative initial infectious and cardiac workup. Based on these findings, we had a high initial suspicion that either COP or IEP was present. Our patient had a bronchoscopy done and was promptly started on steroid therapy soon after, which led to rapid clinical improvement. Pathological specimens were inconclusive, but the patient continued to improve, thereby confirming the presence of either form of ILD. The patient was subsequently discharged home with oxygen and recommended to follow up with a pulmonologist for further outpatient testing and management.

## Introduction

Patients with diffuse interstitial lung diseases (ILD) are often difficult to diagnose. There are several conditions that present clinically with similar symptoms, laboratory findings, and imaging. Cryptogenic organizing pneumonia (COP) and idiopathic eosinophilic pneumonia (IEP) are two such entities that present with identical clinical pictures. Patients with either diagnosis present with respiratory symptoms, ground-glass interstitial opacities, and evidence of consolidation. Both conditions have more peripheral involvement on high-resolution computed tomography (HRCT) [1]. COP is a result of intra-alveolar fibroproliferative processes leading to alveolar injury. Unlike other forms of ILD, this fibrotic process is completely reversible with steroid therapy [2]. IEP, however, is thought to be a hypersensitivity reaction to a triggering agent in the lung. This agent can be non-specific and leads to localized alveolar inflammation prompted mainly by eosinophilic activation and endothelial injury [3]. Infectious and autoimmune workups are negative in both cases, and patients respond dramatically to steroid therapy over a short period of time [2-3]. In this case report, we present a patient with the clinical symptoms of ILD and attempt to identify either COP or IEP.

## Case presentation

A 50-year-old African American male with a pertinent history of hypertension and 20 pack-years of smoking came to our hospital with initial complaints of respiratory distress and hypoxia for the last two days. The patient had been having progressive dyspnea for three weeks prior to presentation but noted a marked decline on the day he came into the ER. The patient did not have a history of coronavirus disease 2019 (COVID-19) and was unvaccinated against COVID-19.

On examination, the patient had diffuse crackles bilaterally that were more prominent in the lung bases. He was also having pursed lip breathing with some use of accessory lung muscles initially and was placed on supplemental oxygen at 15 L/min. Laboratory testing is shown in Table 1.

**Table 1:** Laboratory findings on hospital day 1 NT-proBNP: N-terminal pro–B-type natriuretic peptide; pCO2: partial pressure of carbon dioxide; pO2: partial pressure of oxygen; HCO3: bicarbonate

Blood work	Patient's result	Normal Range	Interpretation
White Blood Cell Count	13.0 K/UL	4 - 11 K/UL	High
Eosinophil Count	0.74 K/UL	0 - 0.60 K/UL	High
Hemoglobin	11.7 g/dL	13.5 - 17.0 g/dL	Low
Creatinine	1.00 mg/dL	0.60 - 1.50 mg/dL	Normal
NT-proBNP	44 pg/mL	≤ 125 pg/mL	Normal
Lactate Level	2.4 mmol/L	0.5 - 1.7 mmol/L	High
Arterial Blood Gas (on 15L oxygen)			
pH	7.49	7.35-7.45	High
pCO2	45 mmHg	35 - 45 mmHg	Normal
pO2	53 mmHg	76 - 100 mmHg	Low
HCO3	34 mmol/L	22 - 28 mmol/L	High

The patient was started on bilevel positive airway pressure (BiPAP) with inspiratory positive airway pressure (IPAP) of 12, expiratory positive airway pressure (EPAP) of 6, and 100% fraction of inspired oxygen (FiO2). Despite this, his blood gas revealed a partial pressure of oxygen (pO2) of 76, confirming the presence of ARDS (arterial oxygen pressure (PaO2)/FiO2 ratio = 76). The patient remained on this for the first 24 hours and was then switched to a hi-flow nasal cannula with settings of 60L at 100% FiO2 to achieve oxygen saturation > 90%. An urgent chest CT angiogram was performed, which revealed bilateral interstitial infiltrates that were more prominent in the peripheral lung fields. Due to his sepsis picture with possible pneumonia, the patient was started on IV ceftriaxone 1 gm IV daily and azithromycin 500 mg IV daily and was admitted to the intensive care unit.

Subsequent infectious disease workup, including blood and sputum cultures, coccidioidomycosis testing, legionella testing, pneumococcal testing, and mycoplasma testing, all returned negative. A transthoracic echocardiogram revealed only grade 1 diastolic dysfunction without any significant pulmonary hypertension or valvular abnormalities. Autoimmune testing, including antinuclear antibody (ANA), complement, rheumatoid factor, anti-dsDNA, and anti-SSA/B were also negative. Despite continued antibiotic therapy, the patient continued to require a hi-flow nasal cannula of 60L at 80% and was not clinically improving. Repeat laboratory findings showed an improvement of the WBC count to 9.4 K/uL as well as a normal procalcitonin despite a poor clinical picture.

**Figure 1:**
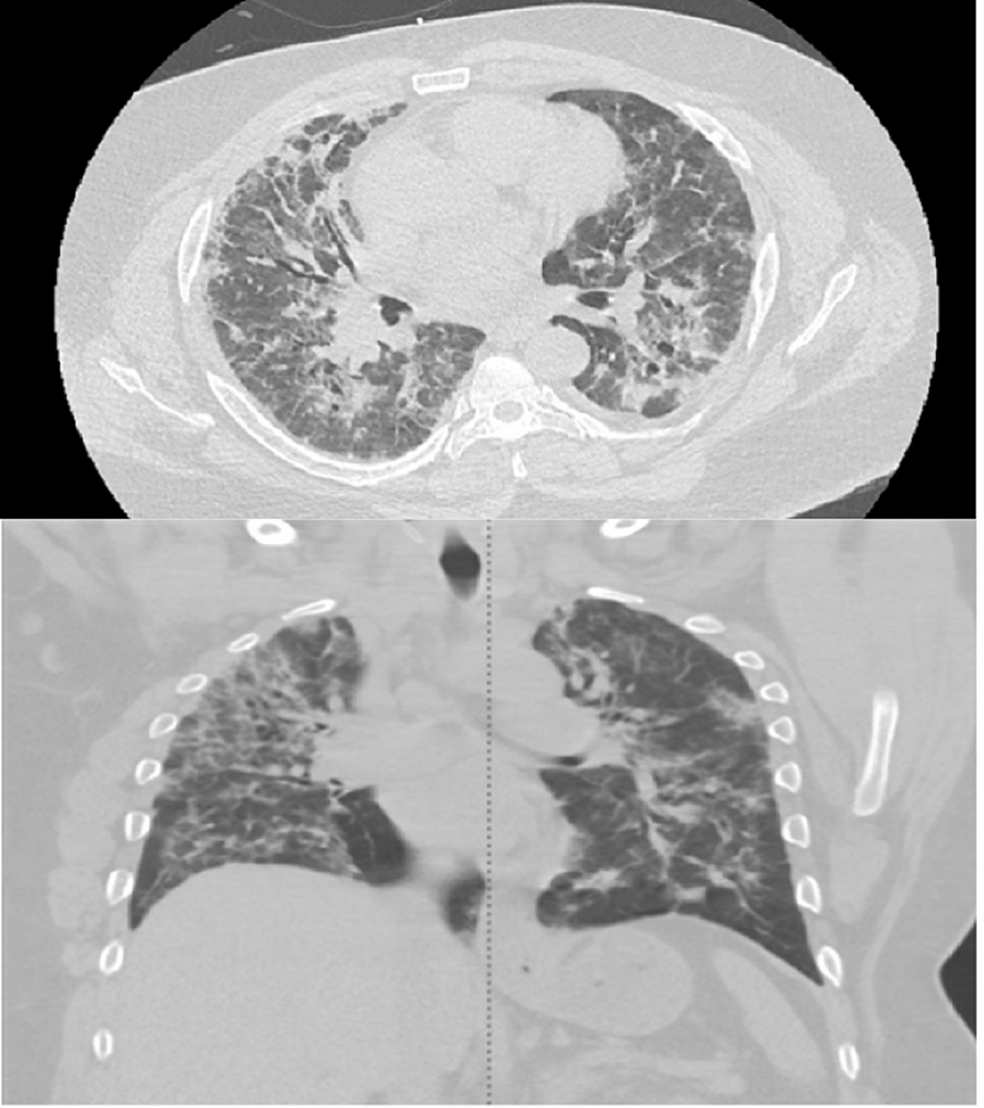
Axial lung window CT image and coronal CT lung window image demonstrate peripheral areas of consolidation, with faint adjacent areas of ground glass opacification The findings are nonspecific and cryptogenic organizing pneumonia and idiopathic eosinophilic pneumonia remain in the differential radiologically.

On hospital Day 5, the patient had a high-resolution chest CT, which confirmed bilateral patchy consolidations that were more prominent in the peripheral lung fields (Figure 1). Bronchoscopy was also performed with bronchoalveolar lavage (BAL) washings showing an eosinophil predominance of 15%. A transbronchial biopsy was also obtained, which showed chronic inflammation without organizing features. As clinical suspicion was high for either COP or IEP based on classical CT findings and negative infectious workup, the patient was started on high-dose IV methylprednisolone at 60 mg every six hours.

**Figure 2:**
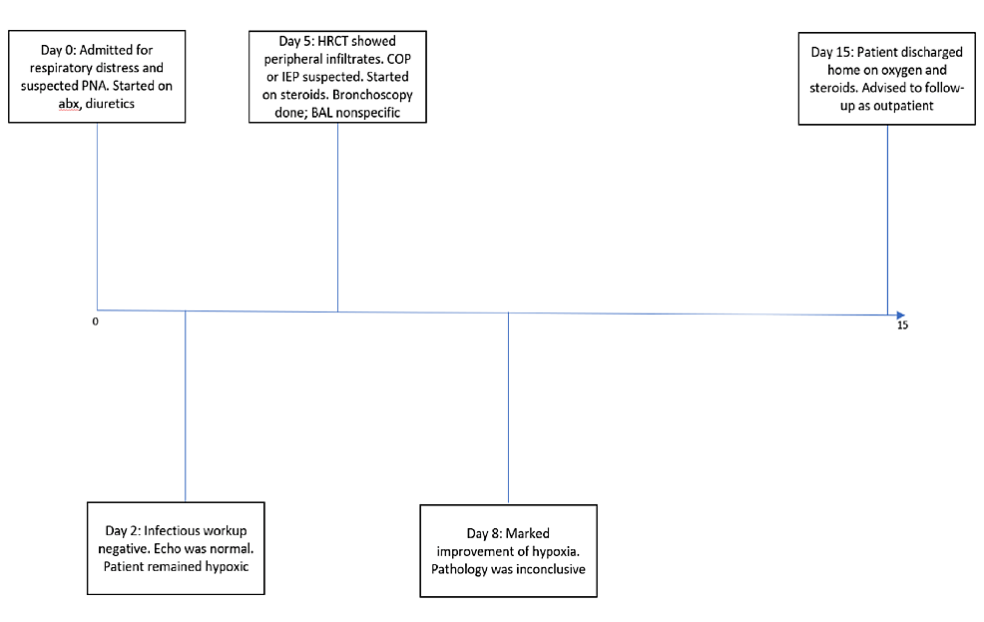
A timeline of the hospital course of the presented case

With this treatment, the patient had a dramatic improvement in his symptoms over the next few days. By hospital day 8, the patient had been weaned down to 10 L/min via nasal cannula. The patient was continued on a slow steroid taper over the next week as his oxygen requirements gradually improved. He was discharged to home on hospital day 15, requiring only 4 L/min of oxygen via nasal cannula. Unfortunately, the details after discharge are not available due to loss to follow-up (see Figure 2 for timeline).

## Discussion

The clinical triad of rapidly progressive hypoxia, bilateral interstitial infiltrates, and response to steroids is classical of idiopathic interstitial lung diseases [4]. Add a peripheral predominance of infiltrates and eosinophilia, and you narrow this further down to COP and IEP. The distinguishing features of COP are the localization of infiltrates in the bases and subpleural regions along with mass-like lesions in some cases while IEP patients have an upper lung zone distribution and more ground glass features [1,5]. Eosinophilia is also more common in IEP and generally becomes more apparent as the hospital course progresses [3]. On pathology, the classical organizing pattern of COP presents as areas of granulation tissue in the alveoli and terminal bronchioles [6].

In contrast, IEP presents as eosinophilic infiltration of the alveolar spaces with abscess formation in some cases [3,5,7]. Notably, pathology in both cases can be altered if treated with steroids before sampling. Patients with COP do require a prolonged course of steroids and often have a recurrence of symptoms [8] while patients with acute IEP show complete resolution at the end of their steroid treatment [9].

Our patient presented with these exact findings and had some clinical features consistent with both entities. He had rapidly progressive hypoxia with an ARDS picture and did not respond initially to antibiotic therapy. He was noted to have eosinophilia on presentation, which favors IEP over COP but did not have the typical persistence of eosinophilia over his hospital course. He did have the classical interstitial pattern on HRCT with more peripheral infiltrates, but they were diffuse and involved the entirety of both lung fields. Of note, there were no mass-like lesions to biopsy in this case. BAL lavage specimens did show a predominance of eosinophilia, which favors IEP, but it did not meet the classical threshold of ≥ 25% which is noted in the literature. Pathological specimens were indeterminate for any specific diagnosis, but the sample obtained was small and could have missed critical areas. The patient also had clinical improvement with steroids but was lost to follow-up; hence, the possibility of recurrence was not established.

Both COP and IEP have very similar incidence rates, especially among males of this age group. The presentation of our patient suggests that establishing ILD is possible at an early stage of presentation, especially in those patients with ARDS, a peripheral predominance of interstitial infiltrates, and eosinophilia. In these cases, the clinicians should exercise a low threshold to initiate high-dose steroid therapy due to its benefits in overall prognosis. This can be initiated concurrently with antibiotic therapy that will counteract the harmful effects of a missed bacterial infection. Any large mass-like lesions should undergo urgent biopsy prior to initiation of steroids to establish a pathological diagnosis. Pulmonary resection can also be a consideration, especially in patients with suspected COP, as it is both diagnostic and curative in those with discrete lesions [10]. However, this practice has mostly fallen out of favor.

## Conclusions

Differentiating between COP and IEP is challenging, as both sets of patients present identically and are treated similarly. Pathological diagnosis is challenging unless there are obvious discrete lesions, as more aggressive measures, including transthoracic biopsies and surgical resections, have fallen out of favor. This case report suggests that patients who present with clinical findings suggestive of either COP or IEP should receive prompt corticosteroid therapy due to its dramatic improvement in symptoms regardless of the pathological diagnosis.
